# MUC1‐C drives myeloid leukaemogenesis and resistance to treatment by a survivin‐mediated mechanism

**DOI:** 10.1111/jcmm.13662

**Published:** 2018-05-15

**Authors:** Dina Stroopinsky, Hasan Rajabi, Myrna Nahas, Jacalyn Rosenblatt, Maryam Rahimian, Athalia Pyzer, Ashujit Tagde, Akriti Kharbanda, Salvia Jain, Turner Kufe, Rebecca K. Leaf, Eleni Anastasiadou, Michal Bar‐Natan, Shira Orr, Maxwell D. Coll, Kristen Palmer, Adam Ephraim, Leandra Cole, Abigail Washington, Donald Kufe, David Avigan

**Affiliations:** ^1^ Beth Israel Deaconess Medical Center Harvard Medical School Boston MA USA; ^2^ Harvard Medical School Dana Farber Cancer Institute Boston MA USA

**Keywords:** acute myeloid leukaemia, MUC1, survivin

## Abstract

Acute myeloid leukaemia (AML) is an aggressive haematological malignancy with an unmet need for improved therapies. Responses to standard cytotoxic therapy in AML are often transient because of the emergence of chemotherapy‐resistant disease. The MUC1‐C oncoprotein governs critical pathways of tumorigenesis, including self‐renewal and survival, and is aberrantly expressed in AML blasts and leukaemia stem cells (LSCs). However, a role for MUC1‐C in linking leukaemogenesis and resistance to treatment has not been described. In this study, we demonstrate that MUC1‐C overexpression is associated with increased leukaemia initiating capacity in an NSG mouse model. In concert with those results, MUC1‐C silencing in multiple AML cell lines significantly reduced the establishment of AML in vivo. In addition, targeting MUC1‐C with silencing or pharmacologic inhibition with GO‐203 led to a decrease in active β‐catenin levels and, in‐turn, down‐regulation of survivin, a critical mediator of leukaemia cell survival. Targeting MUC1‐C was also associated with increased sensitivity of AML cells to Cytarabine (Ara‐C) treatment by a survivin‐dependent mechanism. Notably, low *MUC1* and *survivin* gene expression were associated with better clinical outcomes in patients with AML. These findings emphasize the importance of MUC1‐C to myeloid leukaemogenesis and resistance to treatment by driving survivin expression. Our findings also highlight the potential translational relevance of combining GO‐203 with Ara‐C for the treatment of patients with AML.

**Key Points**

- MUC1‐C is essential for AML establishment in an NSG mouse model.
- Targeting MUC1‐C increases sensitivity of AML cells to cytarabine by down‐regulating survivin.

## 1 INTRODUCTION

Acute myeloid leukaemia (AML) is a lethal haematological malignancy characterized by the emergence of a clonal population of primitive myeloid cells that exhibit a pattern of dysregulated growth and self‐renewal.^1^ Responses to cytotoxic agents, such as Cytarabine (Ara‐C) and daunorubicin, are often observed in patients with AML; however, disease relapse is common because of the emergence of chemotherapy‐resistant disease.^2^ In this context, progression of AML is associated with genetic and epigenetic changes that promote aggressiveness and resistant disease. There is a significant need to identify critical pathways that govern leukaemic progression and offer potential targets for novel therapeutics.

Mucin 1 (MUC1) is a heterodimeric protein that is aberrantly expressed in cancer cells, including AML blasts. The oncogenic MUC1‐C subunit drives critical hallmarks of malignant cells, including cell proliferation, resistance to apoptosis, self‐renewal, and tissue invasion.^3^, ^4^, ^5^, ^6^ Intriguingly, we have demonstrated that MUC1 is uniquely expressed by AML stem cells as compared to normal haematopoietic stem cells.^7^ Primary AML cells expressing high levels of MUC1 efficiently induce leukaemic engraftment in a xenogeneic murine model, whereas MUC1 low expressing cells isolated from bone marrow of patients with active AML engraft normal haematopoietic elements.

The oncogenic function of the transmembrane MUC1‐C subunit is dependent on the formation of homodimers, which are required for translocation to the nucleus and interactions with downstream effectors.^8^, ^9^, ^10^, ^11^ The MUC1‐C cytoplasmic domain is phosphorylated by c‐Src and receptor tyrosine kinases, and interacts with effectors, such as β‐catenin and NF‐κB, that have been linked to transformation. In AML, MUC1‐C associates with the β‐catenin/TCF4 complex, which regulates cell proliferation and differentiation.^5^, ^12^ Accumulation of β‐catenin in the cytoplasm promotes its translocation to the nucleus as a cofactor for transcription factors of the T‐cell factor (TCF) family and activates the transcription of Wnt/β‐catenin target genes. MUC1‐C facilitates the nuclear translocation of dephosphorylated active β‐catenin that is necessary for inducing the expression of cyclin D1, MYC and survivin, a negative regulator of apoptosis.^13^, ^14^, ^15^ Survivin also plays a role in the proliferation and survival of leukaemia induced by the internal tandem duplication of FLT3.^16^ Moreover, survivin is highly expressed in AML progenitor cells and is predictive of poor clinical outcomes in patients with AML.^17^

In this study, we demonstrate that MUC1‐C signalling is critical for leukaemia progression and sensitivity to the cytotoxic agent Ara‐C by a survivin‐mediated mechanism. These findings emphasize the importance of MUC1‐C as a target in AML and support targeting of MUC1‐C with GO‐203 in combination with Ara‐C for the treatment of patients with AML.

## 2 MATERIALS AND METHODS

### 2.1 AML patient derived cells and cell lines

AML cell lines THP1, MV4‐11 and MOLM‐14 were purchased from ATCC and cultured in RPMI 1640 media (Cellgro, Manassas, VA) supplemented with heat‐inactivated 10% Fetal Bovine Serum (Sigma, St. Louis, MO) and 100 IU/mL penicillin, and 100 μg/mL streptomycin (Cellgro, Manassas, VA). MOLM‐14 and THP1 cells were transduced with a lentiviral vector expressing a MUC1 shRNA (MUC1shRNA; Sigma) or with a scrambled control shRNA vector (CshRNA; Sigma).^18^ Alternatively, MUC1 knockdown was achieved using CRISPR/Cas9 technology as described.^19^ For overexpression of MUC1‐C, cells were transduced with lentiviruses expressing pHR‐CMV‐GFP (vector) or MUC1‐C (MUC1‐C). Primary cells were transduced with help of Transdux transduction reagent (System Biosciences Cat# LV850A‐1) using a previously concentrated viral particles, which were obtained with help of Lenti‐X concentrator (clontech Cat #631231). For qRT‐PCR analysis the total RNA was isolated from above transduced cells, using RNAeasy kit (Qiagen Cat#74104) and cDNA synthesis was done with 2 μg of total RNA using High Capacity RNA transcription kit (Thermo Fisher Cat#4368814). The cDNA was diluted further for Real Time PCR using SYBR green master mix (Applied Biosystem Cat#4368708) in applied biosciences Real time PCR machine (AB6000). For the CRISPR edited cell line, sgRNAs targeting the first exon of the MUC1 gene were cloned into a lenti‐plasmid (Genome Engineering Production Group, Harvard Medical School). MOLM‐14 cells were transduced with viral vector containing the lenti‐CRISPR plasmid and successfully transduced clones were selected for by limiting dilution and maintained in 2 μg/mL Puromycin (Sigma, St. Louis, MO). MCF‐7 breast cancer cell line was used as positive control for MUC1 expression.

Bone marrow aspirates samples were obtained from patients with newly diagnosed AML as per an institutionally approved protocol. Mononuclear cells were isolated by ficoll density centrifugation. For assessment of active β‐catenin, CD34+ cells were isolated using the MiniMacs CD34 cell isolation kit (Miltenyi Biotec). The bulk AML cell population was used for in vivo experiments. Cells were treated with the MUC1‐C inhibitor, GO‐203, and as a control, the CP‐2 peptide.^8^

### 2.2 Quantitative RT PCR

Quantitative real‐time (RT)‐PCR was performed on cDNA synthesized from total cell RNA using the Thermoscript RT‐PCR system (Invitrogen). The SYBR green qPCR assay (Applied Biosystems) was used with diluted cDNA. The samples were amplified with the ABI Prism 7000 Sequence Detector (Applied Biosystems). Forward and reverse primers are Survivin Fwd (5′‐ TAATACCAGCACTTTGGGAGG‐3′) Rev (5′‐ GGCTCTTTCTCTGTCCAGTTTC‐3′). MUC1 Fwd (5′‐ TACCGATCGTAGCCCCTATG ‐3′), Rev (5′‐CTCACCAGCCCAAACAGG‐3′) and GAPDH Fwd (5′‐ CCATGGAGAAGGCTGGGG‐3′) Rev (5′‐ CAAAGTTGTCATGGATGACC‐3′).

### 2.3 Leukaemia engraftment in NSG mice

Bone marrow derived AML cells and AML cell lines were inoculated retro‐orbitally into sublethally irradiated (300 rads) NOD‐SCID IL2Rgammanull (NSG; 6 week old female) mice (Jackson Laboratories). After sacrifice, bone marrow and spleen cells were harvested and red blood cells (RBC) were removed using RBC lysis buffer (Sigma). Human AML engraftment was detected by staining cells with PE‐conjugated anti‐hCD45 and, as a control, FITC‐conjugated antimouse mCD45. In certain experiments, the cells were also analysed for hCD34, hCD11C, hCD19 or hCD20 by multichannel flow cytometry using CellQuest, Diva or Kaluza software. In order to confirm AML blast morphology, cytospins were made from BM cells. The cells were then fixed in methanol and stained using the standard Wright Giemsa protocol. The cells were visualized with contrast light microscopy (Olympus AX70 microscope) using an oil immersion objective lens (×100).

### 2.4 Immunoblot analysis

Cell lysates were prepared as described.^20^ Soluble proteins were analysed by immunoblotting with anti‐MUC1‐C (ThermoScientific), anti‐active β‐catenin (Cell Signaling Technologies), anti‐survivin (Cell Signaling Technologies) and anti‐β‐actin (Sigma). Antigen‐antibody complexes were visualized by enhanced chemoluminescence (ECL; Amersham Biosciences). Densitometry analysis was performed used image J software.

### 2.5 Analysis of intracellular protein expression by flow cytometry

*For* β*‐catenin expression* ‐ isolated leukaemia CD34+ progenitors were permeabilized with a saponin‐based reagent (eBioscience). The cells were then stained with purified anti‐active β‐catenin (Millipore) for 1 hour followed by secondary labelling of the cells with FITC‐conjugated goat antimouse IgG and then analysis by flow cyometry. *For survivin expression* ‐ AML cells underwent fixation and permeabilization using Transcription Factor Staining Buffer Set (eBioscience) and then stained with 0.5 μg PE‐conjugated anti‐survivin STLALYV monoclonal antibody (Thermo Fisher). *For Ki67 expression* ‐ AML cells were permeabilized with a saponin‐based reagent (eBioscience). The cells were then incubated with Pacific Blue‐conjugated anti‐Ki67 monoclonal antibody (BioLegend) at room temperature in the dark for 30 minutes. Purified Mouse IgG1, κ was used as isotype control. The cells were then analysed using the Gallios flow cytometer.

### 2.6 Cytotoxicity assays

AML cells were seeded in white flat‐bottom 96‐well plates at 10 000 cells/well. At 48 hours of treatment, cell viability was assessed using the CellTiter‐Glo^®^ (CTG) Luminescent Cell Viability Assay. Raw luminescence values were obtained from each well using Infinite M200 Pro luminometer (Tecan). Drug synergy was assessed using CompuSyn software program in which combination index (CI) <0.7 considered as synergistic and >0.7 considered as antagonistic. In addition, dead cells were detected by addition of 0.1 mg/mL propidium iodide (PI) and apoptotic cells were detected by Annexin V (FITC) apoptosis detection kit (BD Biosciences) using flow cytometry.

### 2.7 Microarray gene expression data

Gene expression and clinical data were analysed for previously described cohort of adult AML patients: dataset of 260 patients with diverse cytogenetic and molecular abnormalities described by Valk et al Gene expression profiles of AML patients were downloaded from NCBI GEO dataset (https://www.ncbi.nlm.nih.gov/geo, accession number GSE1159). Probe intensity values were normalized using the bioconductor “affy” package using R version 3.3.1 for the probe of interest. The normalization is based on Affymetrix MAS5.0 with the absolute scale factor (sc) of 100. Patients were stratified dichotomously based on an optimal threshold of *MUC1* and *BIRC5* expression. Overall survival of both low and high expression groups were examined using “survival” package using R version 3.3.1.

### 2.8 Statistical analysis

Data of two tested groups were compared using the Student's *t*‐test. *P*‐Values less than. 05 were considered significant.

## 3 RESULTS

### 3.1 MUC1‐C overexpression leads to increased leukaemogenicity in NSG mice

**Figure 1:**
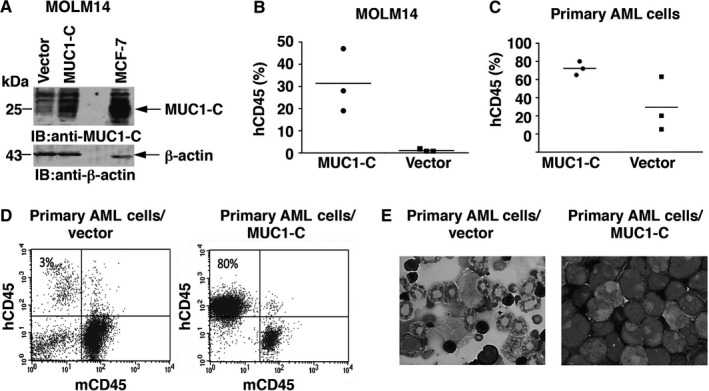
MUC1‐C overexpression leads to increased leukaemogenicity in NSG mice. MUC1‐C was overexpressed in MOLM14 cells. A, The cells were harvested and lysates were immunoblotted for the expression of MUC1‐C using anti‐CT2 monoclonal antibody. MCF7 cells were used as positive control. The cells were then inoculated into sublethally irradiated NSG mice at a low dose of 1000 cells/mouse. 21 d post inoculation the mice were killed and BM cells were isolated and analysed for human CD45 expression. B, The results are expressed as percentages of hCD45+ leukaemia cells in the BM of individual mice inoculated with AML cells with MUC1‐C overexpression and control vector. The horizontal bar represents the mean percentage of hCD45+ cells (n = 3; *P* < .05). C, MUC1‐C was overexpressed in AML cells obtained from a BM aspirate of a patient with AML. The cells were then inoculated into sub‐lethally irradiated NSG mice 5 × 10^5^ cells/mouse. Ninety days post inoculation the mice were killed and BM cells were isolated and analysed for human CD45 expression. The results are expressed as percentages of hCD45+ leukaemia cells in the BM of individual mice inoculated with AML cells with MUC1‐C overexpression and control vector. The horizontal bar represents the mean percentage of hCD45+ cells (n = 3; *P* < .05). D, Representative FACS plots of mice inoculated with MUC1‐C overexpressed and control patient derived AML cells. E, Cytospins were prepared from bone marrow cells isolated from mice inoculated with MUC1‐C overexpressed AML cells and control cells. The cytospins were then stained using standard Giemsa staining protocol. BM morphology of representative mice is shown

To investigate the role of MUC1‐C in leukaemia induction in vivo, MUC1‐C was stably overexpressed in MOLM‐14 AML cells as compared to that in cells transduced with GFP vector (Figure 1A). MOLM‐14/MUC1‐C and MOLM‐14/vector cells were inoculated into sublethally irradiated NSG mice at 1000 cells/mouse, a dose characteristically insufficient to support rapid AML engraftment. At 21 days following inoculation, the mice were killed and bone marrow (BM) cells were analysed for the engraftment of human CD45+ AML cells. The results demonstrated significantly greater engraftment of human CD45+ cells in mice inoculated with MOLM‐14/MUC1‐C versus MOLM‐14/vector cells (Figure 1B) with mean levels of AML engraftment of 31.1% and 1% respectively (n = 3; *P* = .02). MUC1‐C was also overexpressed in primary AML cells isolated from bone marrow of AML patient at diagnosis. NSG mice were inoculated with 5 × 10^5^ AML/MUC1‐C or AML/vector cells. The mouse bone marrow cells were analysed 90 days following inoculation and showed hCD45+ cells engraftment of 72% and 29% for AML/MUC1‐C and control AML/vector cells respectively (Figure 1C, D). Furthermore, cytospins prepared from bone marrow cells of mice inoculated with AML/MUC1‐C cells showed monomorphic blast cells consistent with AML. In contrast, bone marrow cells isolated form mice inoculated with AML/vector cells demonstrated normal mouse bone marrow cell morphology typical to NSG mice, and no evidence of human AML engraftment (Figure 1E). Of note, MUC1‐C overexpression did not lead to increase in expression of proliferation marker Ki67 (Figure S2A) in MOLM14 AML cells.

### 3.2 MUC1‐C silencing in AML leads to loss of leukaemia initiating capacity in NSG mice

**Figure 2:**
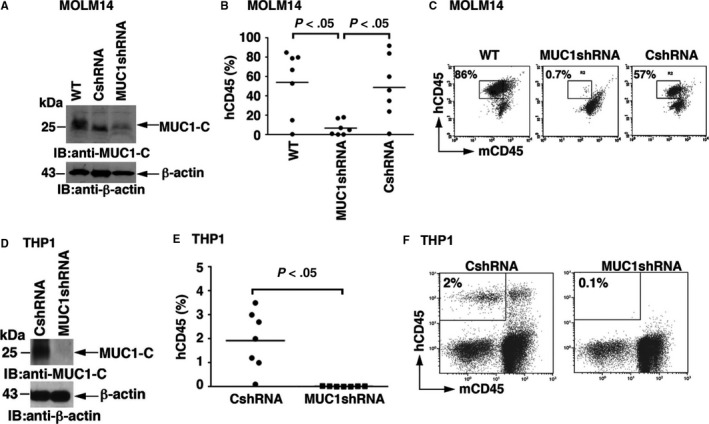
MUC1‐C silencing in MOLM14 and THP1 cells leads to loss of leukaemia initiating capacity in NSG mice. MUC1‐C was silenced in MOLM‐14 and THP1 AML cells using lentiviral infection with a shRNA hairpin sequence against MUC1‐C. Wild‐type MOLM‐14 (wt) cells and MOLM‐14 cells infected with control shRNA (control) were used as controls. A, MOLM14 cells were harvested and lysates were immunoblotted for the expression of MUC1‐C using anti‐CT2 monoclonal antibody. The cells were then inoculated into sub‐lethally irradiated NSG mice 10 × 10^3^/mouse. 14 d post inoculation, the mice were killed and BM cells were isolated and analysed for hCD45 expression. (B) The results are expressed as percentages of hCD45+ leukaemia cells in the BM of individual mice in the MUC1 silenced and the control groups. The horizontal bar represents the mean percentage of hCD45+ cells. C, FACS plots of BM cells analysed for AML engraftment of representative mice. MUC1‐C was silenced in THP1 AML cells using lentiviral infection with a shRNA hairpin sequence against MUC1‐C. THP1 cells infected with control shRNA were used as controls. D, The cells were harvested and lysates were immunoblotted for the expression of MUC1‐C using anti‐CT2 monoclonal antibody. E, THP1 cells were inoculated into sub‐lethally irradiated NSG mice 500 × 10^3^/mouse. 30 d post inoculation the mice were killed and BM cells were isolated and analysed for hCD45 expression. The results are expressed as percentages of hCD45+ leukaemia cells in the BM of individual mice in the MUC1 silenced and the control groups. The horizontal bar represents the mean percentage of hCD45+ cells. One of three independent experiments is shown. F, FACS plots of BM cells analysed for AML engraftment of representative mice

To further study the significance of MUC1‐C expression on engraftment of AML in vivo, MUC1‐C was silenced in MOLM‐14 cells using a lentiviral shRNA hairpin against MUC1‐C (MUC1shRNA). MOLM‐14 cells were also infected with a control shRNA (CshRNA) (Figure 2A). MOLM‐14/WT, MOLM‐14/MUC1shRNA and MOLM‐14/CshRNA cells (10 × 10^3^ cells/mouse) were injected retro‐orbitally into sublethally irradiated NSG mice in cohorts of eight mice/group. The animals were killed at day 14 following inoculation for analysis of leukaemia establishment. The mice inoculated with MOLM‐14/WT cells and MOLM‐14/CshRNA cells developed massive bone marrow infiltration with leukaemia cells that was observed in 7/8 of recipient mice (Figure 2B). Flow cytometric analysis of the bone marrow of mice revealed mean involvement of 54% and 48% with human CD45+ leukaemia cells in the MOLM‐14/WT and MOLM‐14/CshRNA groups respectively (Figure 2C). Remarkably, the 8 mice injected with MOLM‐14/MUC1shRNA cells showed minimal evidence of leukaemic engraftment (Figure 2C). Thus, analysis of these mice revealed mean AML involvement of only 6% of the bone marrow cells, which was significantly lower than that observed in the MOLM‐14/WT and MOLM‐14/CshRNA groups (*P* = .003 and *P* = .01 respectively; Figure 2C). Interestingly, MUC1 silencing led to a significant decrease in Ki67 expression in MOLM‐14/MUC1shRNA cells compared to control (Figure S2B) indicating decrease in proliferative capacity.

To confirm these findings, NSG mice were similarly inoculated with THP1 cells stably expressing the MUC1shRNA or the CshRNA (Figure 2D). In 3 independent experiments, a significant decrease in leukaemic engraftment was observed for THP1/MUC1shRNA, as compared to THP1/CshRNA, cells (Figure 2E, F).

### 3.3 MUC1 silencing leads to reduced nuclear translocation of β‐catenin and decreased survivin expression

To evaluate the mechanism by which MUC1‐C‐mediated signalling promotes leukaemogenicity, we assessed the effect of silencing MUC1‐C on downstream effectors important for leukaemic survival. Specifically, we examined the effect of MUC1‐C silencing on the expression of survivin, a target gene of the WNT/β‐catenin/TCF pathway, which is critical for leukaemia survival, resistance to apoptosis and disease progression.^14^, ^16^, ^17^, ^21^, ^22^, ^23^, ^24^, ^25^, ^26^, ^27^, ^28^, ^29^, ^30^

**Figure 3:**
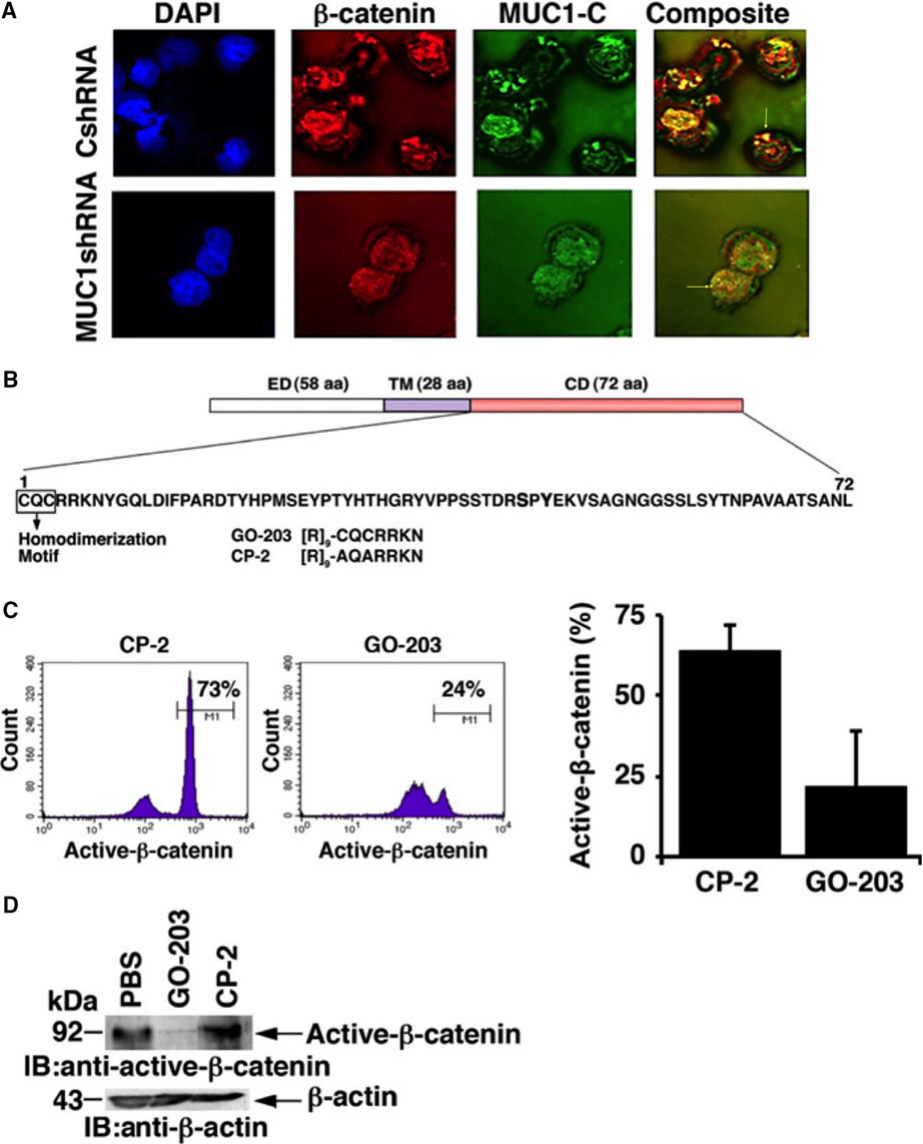
MUC1 silencing leads to reduced β‐catenin levels and its decreased translocation to the nucleus. A, MUC1‐C was silenced in MOLM‐14 AML cells using lentiviral shRNA hairpin against MUC1‐C. As a control, MOLM ‐14 cells were infected with control shRNA. For immunofluorescence evaluation of co‐locolization of MUC1 and β‐catenin to the nucleus, cytospins of MUC1‐C silenced and control MOLM‐14 cells were prepared and stained with antibodies against β‐catenin (red) and MUC1‐C (green). DAPI was used to visualize nuclei (blue). Images were acquired with a confocal Microscope (n = 3). B, Schematic representation of the MUC1‐C subunit. MUC1‐C consists of a 58 aa non‐shed extracellular domain (ED), a 28 aa transmembrane domain (TM) and a 72 aa cytoplasmic domain (CD). The MUC1‐C cytoplasmic domain contains a CQC motif that is necessary for MUC1‐C homodimerization, localization to the nucleus and oncogenic function. The MUC1‐C CQC motif is the target of the GO‐203 inhibitor. C, CD34+ cells isolated from three patients with AML were treated with 2.5 μmol/L GO‐203 MUC1‐C inhibitor or CP‐2 control every 24 h for 3 d. Treated cells were harvested and analysed by flow cytometry for active dephosphorylated β‐catenin expression as shown in a representative experiment and summary of 3 independent experiments (mean ± SD). D, Lysates were immunoblotted for the expression of active β‐catenin using an anti‐dephospho‐β‐catenin monoclonal antibody. β‐actin was used as a control. A representative example of each is shown (n = 3)

Analysis of MOLM‐14/CshRNA cells using immunoflourescent imaging demonstrated co‐localization of MUC1‐C and β‐catenin in the nucleus. In contrast, silencing MUC1‐C in MOLM‐14/MUC1shRNA cells was associated with a significant decrease in nuclear localization of MUC1‐C and β‐catenin (Figure 3A).

The MUC1‐C cytoplasmic domain contains a CQC motif that is necessary and sufficient for MUC1‐C homodimerization and function^31^, ^32^, ^33^ (Figure 3B). Accordingly, we developed a cell‐penetrating peptide, designated GO‐203, which interacts with the CQC motif and blocks MUC1‐C homodimerization^32^, ^33^ (Figure 3B). The inactive CP‐2 peptide was synthesized as a control^32^, ^33^ (Figure 3B). Notably and in concert with the function of MUC1‐C in stabilizing β‐catenin,^12^ we found that targeting MUC1‐C with GO‐203 in CD34+ AML cells is associated with marked down‐regulation of active β‐catenin as determined by flow cytometry (Figure 3C) and immunoblotting (Figure 3D).

**Figure 4:**
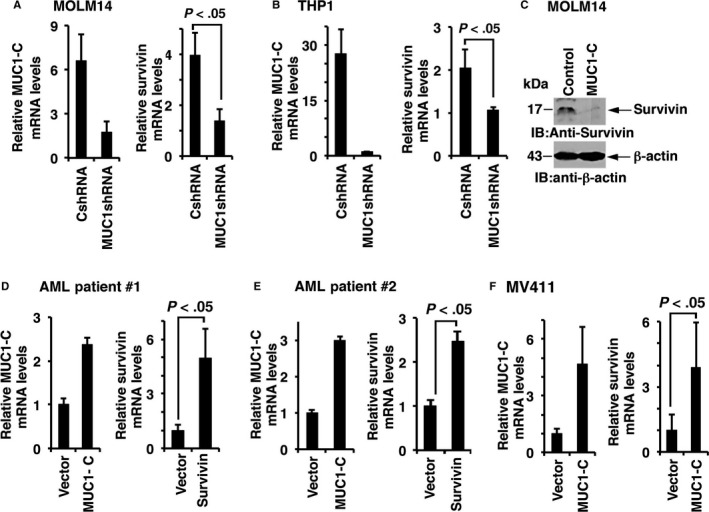
MUC1‐C levels regulate survivin expression. MUC1‐C was silenced in MOLM‐14 and THP1 AML cells a using lentiviral shRNA hairpin against MUC1‐C. As a control, the cells were infected with control shRNA. (A,B) A representative experiment showing MUC1‐C and survivin mRNA levels, evaluated using qPCR. Each condition was performed in triplicate. (C) MOLM14 cells were harvested and lysates were immunoblotted for the expression of survivin using anti‐survivin monoclonal antibody (n = 2). (D,E) MUC1‐C was overexpressed in bone marrow cells isolated from patients with AML. Control cells were infected with a vector containing the GFP gene. Survivin expression was evaluated using qPCR (n = 3). (F) MUC1‐C was over‐expressed in MV411 AML cells, control cells were infected with vector containing the GFP gene. MUC1‐C and survivin mRNA levels were evaluated using qPCR. Each condition was performed in triplicate

We then examined the effect of MUC1‐C silencing on the expression of survivin, a downstream target of the β‐catenin/TCF4 pathway. Survivin inhibits caspase activation, thereby leading to the negative regulation of apoptosis and promotion of leukaemia cell survival.^26^ Significantly, MUC1‐C silencing in MOLM‐14/MUC1shRNA and THP1/MUC1shRNA cells resulted in the down‐regulation of survivin expression as demonstrated by decrease in both survivin mRNA (Figure 4A, B) and protein (Figure 4C) levels. In concert with these findings, overexpression of MUC1‐C was associated with increases in survivin mRNA levels as demonstrated in two samples obtained from bone marrows of patients with newly diagnosed AML (Figure 4D, E, Figure S2C) and an additional immortalized AML cell line MV4‐11/MUC1‐C (Figure 4F). Furthermore, flow cytometric analysis of MV4‐11/MUC1‐C cells confirmed increase in survivin expression in the protein level (Figure S2C).

### 3.4 Survivin overexpression in MUC1 silenced AML cells leads to enhanced leukaemia induction

**Figure 5:**
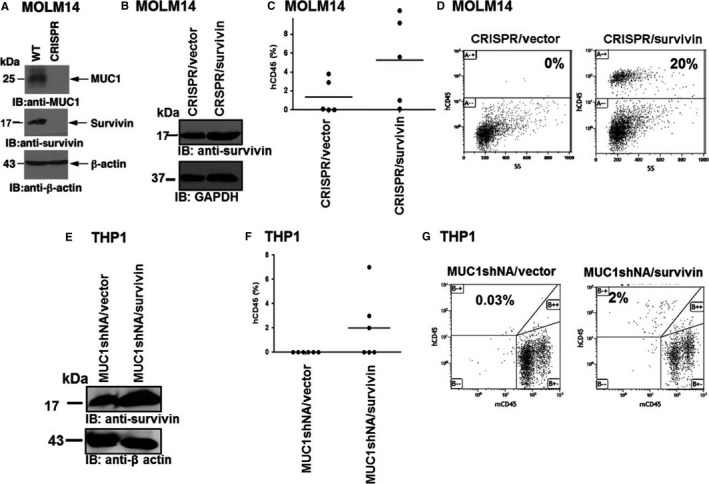
Survivin overexpression in MUC1 silenced AML cells leads to enhanced leukaemia induction. MUC1‐C gene knockdown of the AML cell line, MOLM‐14, was generated using CRISPR/Cas9 technology, MOLM14/WT cells were used as control. (A) Lysates were immunoblotted for the expression of MUC1‐C and survivin, β‐actin was used as a control. Subsequently, GFP tagged survivin or control vector genes were overexpressed in the CRISPR AML cells using lentiviral transduction. GFP+ cells were then isolated using flow cytometric sorting and (B) underwent Western blot analysis for survivin expression with GAPDH used as a control. 10 × 10^3^ cells/mouse were inoculated into NSG mice using retro‐orbital injections. 21 d following inoculation, bone marrow cells were harvested from and analysed for the engraftment of human CD45 AML cells using flow cytometry. (C) The results are expressed as percentages of hCD45+ leukaemia cells in the BM of individual mice in the CRISPR/Survivin group and the control group. The horizontal bar represents the mean percentage of hCD45+ cells. (D) FACS plots of BM cells analysed for AML engraftment of representative mice. MUC1‐C was silenced in THP1 AML cells using lentiviral infection with a shRNA hairpin sequence against MUC1‐C. Subsequently, GFP tagged survivin or control vector genes were overexpressed in these cells using lentiviral transduction. GFP+ cells were then isolated using flow cytometric sorting and (E) underwent Western blot analysis for survivin expression. β‐actin was used as a control. Subsequently, 10 × 10^3^ cells/mouse were inoculated into NSG mice using retro‐orbital injections for 21 d. Bone marrow cells were then analysed for AML engraftment as described above. (F) The results are expressed as percentages of hCD45+ leukaemia cells in the BM of individual mice in the MUC1shRNA/Survivin group and the control group. The horizontal bar represents the mean percentage of hCD45+ cells. (G) FACS plots of BM cells analysed for AML engraftment of representative mice

We next sought to investigate whether the effects of MUC1 silencing on leukaemogenicity is mediated by the down‐regulation of survivin. In these studies, we first investigated MOLM‐14 cells with a knockout of the MUC1 gene using a CRISPR/Cas9 approach (Figure 5A). Consistent with the above findings, down‐regulation of MUC1‐C in the MOLM‐14/CRISPR cells was associated with suppression of survivin expression (Figure 5A). We next overexpressed survivin in the CRISPR cells using lentiviral transduction (Figure 5B, Figure S2D). NSG mice were then inoculated with 10 × 10^3^ MOLM14/CRISPR cells that were transduced with either survivin (CRISPR/survivin) or a control vector (CRISPR/vector). The mice were killed 21 days following inoculation, and the AML burden in the bone marrow was assessed by the detection of human CD45+ cells using flow cytometric analysis. Consistent with our previous results, 2/5 mice showed minimal AML engraftment in the cohort of mice inoculated with MOLM14/CRISPR/vector cells, with mean levels of 1.33% hCD45+ cells in the bone marrow (Figure 5C, D). In contrast, survivin overexpression in MOLM‐14/CRISPR/survivin cells led to increased AML induction with 4/5 mice having detectible disease in the bone marrow and mean levels of 5.3% blast involvement (Figure 5C, D).

These results were confirmed using THP1/MUC1shRNA cells (Figure 5E, Figure S2D). Inoculation of mice with MUC1shRNA/Vector cells did not lead to leukaemia induction. However, in experiments with the MUC1shRNA/surviving cells, 3/6 mice showed AML engraftment with mean levels of 2% blast involvement (Figure 5F, G).

These findings indicate that MUC1 silencing leads to decreases in leukaemogenicity, at least in part by survivin down‐regulation.

### 3.5 MUC1‐C inhibition leads to increased susceptibility to cytarabine via down‐regulation of survivin

**Figure 6:**
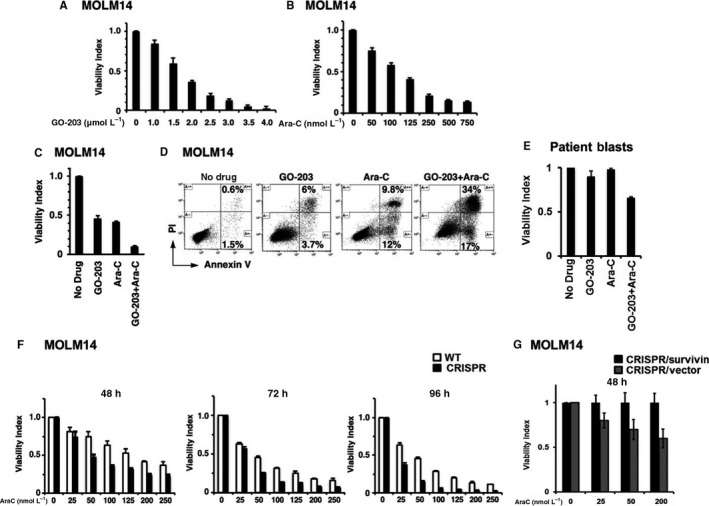
MUC1 inhibition leads to increased susceptibility to cytarabine. MOLM14 AML cells were treated with increasing doses of MUC1 peptide inhibitor, G0‐203, or cytarabine (Ara‐C) for 48 h. (A,B) Cell viability was evaluated using an ATP‐based assay as demonstrated in a representative experiment (n = 3, each experiment performed in triplicates). (C) Cell viability was assessed after 48 h treatment of MOLM14 AML cells with a combination of G0‐203 (2 μmol/L) and Ara‐C (125 nmol/L) as opposed to treatment with each agent alone. (Each experiment was performed in triplicates; n = 3). (D) The results were further confirmed using Annexin/PI evaluation. (E) AML cells were obtained from patients with AML at diagnosis. The cells were then treated with MUC1 peptide inhibitor, G0‐203, or cytarabine (Ara‐C) for 48 h. Cell viability was assessed after 48 h treatment of AML cells with a combination of G0‐203 (2 μmol/L) and Ara‐C (50 nmol/L) as opposed to treatment with each agent alone (each experiment was performed in triplicates; n = 3). (F) The MOLM‐14 CRISPR and wild‐type (WT) cell lines were independently treated with increasing doses of cytarabine. Cell viability was evaluated utilizing an ATP‐based luminescence assay (CTG, Promega) as compared to the MOLM‐14 WT cell line at 48,72 and 92 h after treatment. (G) MOLM‐14/CRISPR cells were transduced with either survivin GFP or control GFP vectors. GFP positive cells were then isolated using flow cytometric sorting and incubated with indicated doses of AraC for 48 h. Cell viability was then assessed as described above

We further sought to determine whether targeting MUC1‐C with the resultant decrease in survivin levels would confer increased susceptibility to Ara‐C. In this way, MOLM‐14 cells were treated with increasing doses of Ara‐C alone, GO‐203 alone or the combination of both agents. Assessment of cell viability after 48 hours demonstrated dose‐dependent cytotoxicity for each agent alone (Figure 6A, B).

Interestingly, exposure of MOLM‐14 cells to the combination of GO‐203 and Ara‐C for 48 hours showed a statistically significant reduction in cell viability compared to that obtained with each agent alone (Figure 6C). These findings were further confirmed using Annexin/PI staining (Figure 6D). Similar results were obtained with MV4‐11 AML cells (Figure S1A‐C), indicating that the GO‐203/Ara‐C combination is synergistic. To further assess this interaction, we employed the combination index (CI) method using CompuSyn to calculate degrees of synergism in drug combinations.^34^, ^35^ Based on the validated CI index, treatment of MOLM‐14 and MV4‐11 cells with GO‐203 in combination with Ara‐C resulted in synergistic killing with CI values of 0.29 and 0.26 respectively.

Furthermore, treatment of tumour cells obtained from three patients with AML at diagnosis using combination of AraC and MUC1 inhibitor GO‐203, led to a similar strong synergistic response (CI 0.28) as demonstrated in representative Figure 6E.

To lend further support for the premise that targeting MUC1‐C is synergistic with Ara‐C, MOLM‐14/WT and MOLM‐14/CRISPR cells were independently treated with increasing doses of Ara‐C. Significantly, the MOLM‐14/CRISPR cells exhibited greater decreases in cell viability as compared to that obtained for MOLM‐14/WT cells at 48, 72 and 96 hours of treatment (Figure 6F), confirming that MUC1‐C targeting significantly increases AML cell susceptibility to Ara‐C.

To examine whether the observed effect of MUC1 inhibition on susceptibility of AML cells to cytotoxic injury is conveyed via survivin‐mediated mechanism, survivin was overexpressed in MOLM‐14/CRISPR AML cells using lentiviral transduction (MOLM‐14/CRISPR/Survivin). MOLM14/CRISPR cells transduced with GFP control vector were used as a control (MOLN‐14/CRISPR/Vector). GFP cells were purified using flowcytometric sorting and then treated with increased doses of AraC. Cell viability was assessed 48 hours after treatment. Indeed, susceptibility of MOLM‐14 cells to Ara‐C following MUC1 silencing was shown to be reversed with survivin overexpression (Figure 6G).

### 3.6 Low *MUC1* and *survivin* expression in human AML is associated with increased overall survival

**Figure 7:**
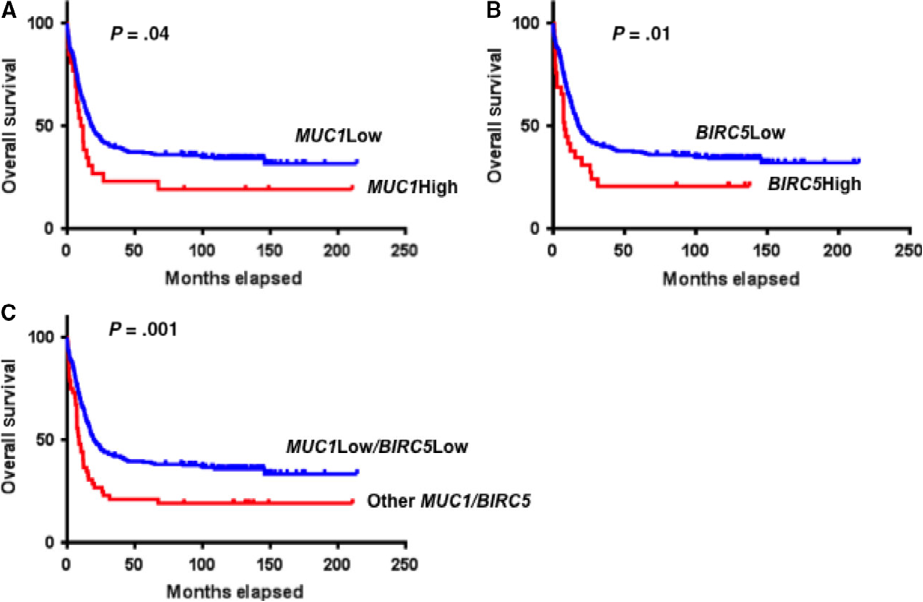
Low *MUC1* and *survivin* Expression in Human AML is Associated with Increased Overall Survival. Overall survival of 260 AML patients with diverse cytogenetic and molecular abnormalities was assessed. A, Patients were stratified into low MUC1 and high MUC1 expression groups based on an optimal threshold (234 low; 26 high) determined by microarray analysis from an independent dataset. The significance measures are based on univariate analysis (*P* = .04). B, Patients were also stratified into low *survivin (BIRC5)* and high *survivin (BIRC5)* expression groups based on an optimal threshold (231 low; 29 high) determined by microarray analysis from an independent dataset. The significance measures are based on univariate analysis (*P* = .01). C, Overall survival was assessed in patients with concomitant low expression of *MUC1* and *survivin* compared to the remainder of AML patients in this group. The threshold was determined based on overlap of previously described single gene expression stratification (52 Low MUC1/Low BIRC5; 208 other MUC1/BIDC5 expressers). The significance measures are based on univariate analysis (0.001)

We hypothesized that increased MUC1 expression on human AML contributes to pathogenesis and predicted that AML with lower expression of *MUC1* would be associated with better clinical outcomes. Consistent with this hypothesis, analysis of previously described group of 260 adult AML patients with diverse cytogenetic and molecular abnormalities^36^ revealed that a dichotomous stratification of patients into low *MUC1* and high *MUC1* expression groups was associated with a significantly decreased risk of death in the low expressing group (*P* = .04) as demonstrated in Figure 7A. Similarly, association of *survivin* (*BIRC5*) expression and clinical outcomes was assessed. The data demonstrated that low survivin expression was correlated with statistically significant prolonged overall survival in this group of AML patients (*P* = .01; Figure 7B). Interestingly, low expression of both *MUC1* and *survivin* led to better clinical outcomes with higher statistical significance (*P* = .001; Figure 1C).

These finding are consistent with previously published survival analysis using TCGA database demonstrating prolonged survival in AML patients with lower *survivin* expression.^37^ Furthermore, our analysis of this database of 168 adult AML patients confirmed that patients with low *MUC1* expression showed significantly prolonged overall survival (*P* = .02, data not shown).

## 4 DISCUSSION

AML is a lethal haematological malignancy characterized by maturation arrest; the capacity for self‐renewal and autonomous cell proliferation, resistance to apoptosis and increased resistance to cytotoxic injury.^1^, ^38^, ^39^, ^40^ AML stem cells are intrinsically resistant to cytotoxic stress because of a low proliferative rate and a multidrug‐resistant phenotype.^41^, ^42^ Clonal plasticity and evolution further results in the emergence of heightened levels of chemotherapy resistance contributing to the nature of disease recurrence following chemotherapy. Defining critical pathways mediating leukaemic transformation, disease evolution and therapeutic resistance is crucial to enhance the biological understanding of the disease and to develop more effective targeted therapies.

MUC1‐C is an oncogenic protein aberrantly expressed in solid tumour and haematological malignancies that is known to promote malignant transformation, tissue invasion, autonomous self‐renewal and resistance to apoptosis.^3^, ^5^, ^8^, ^12^, ^31^, ^43^ MUC1 is overexpressed in majority of patients with AML.^4^, ^7^ Of note, we have demonstrated that MUC1 is uniquely expressed by AML stem cells as compared to normal haematopoietic stem cells. In addition, the subset of cells expressing high levels of MUC1 in primary AML samples are highly efficient in generating leukaemic engraftment in a xenogeneic NSG mouse model,^7^ indicating that MUC1‐C promotes self‐renewal.

In this study, we have demonstrated the importance of MUC1‐C for maintenance of the malignant AML phenotype. Overexpression of MUC1‐C results in marked enhancement of the capacity of human leukaemia cell lines and patient derived primary AML cells to engraft disease in a xenogeneic murine model. Consistent with these findings, silencing of MUC1‐C in AML cells abrogates their capacity for leukaemia induction.

Our studies have also assessed the role of MUC1‐C mediated signalling on the β‐catenin/WNT pathway and its role in inducing survivin, a downstream effector critical for leukaemia biology.^17^ The MUC1‐C cytoplasmic domain has been shown to interact with β‐catenin in several carcinoma cell, but not AML, models.^12^ MUC1‐C associates with the β‐catenin/T‐cell factor 4 (TCF4) complex, which regulates cell differentiation, proliferation and apoptosis.^5^ Accumulation of β‐catenin in the cytoplasm favours its translocation to the nucleus as a cofactor for TCF family transcription factors and thereby activates the transcription of Wnt/β‐catenin target genes.^44^ The MUC1‐C oncoprotein is known to facilitate the nuclear translocation of active β‐catenin to the nucleus, necessary for downstream signalling pathways.^12^, ^45^, ^46^ Here, we have demonstrated that silencing MUC1 in AML cells leads to reduced translocation of β‐catenin to the nucleus. Furthermore, targeting MUC1‐C with GO‐203 led to significantly decreased levels of active β‐catenin in primary CD34+ AML cells.

Survivin (BIRC5), a member of the inhibitor of apoptosis protein (IAP) gene family, inhibits apoptosis, enhances proliferation and promotes angiogenesis.^14^, ^15^, ^16^, ^30^, ^47^ Survivin is highly expressed in AML progenitor cells and is predictive of poor clinical outcomes in patients with AML.^17^ Survivin is thus a critical target in AML that is regulated, at least in part, by the β‐catenin/TCF4 complex.^48^ Our data demonstrate that silencing MUC1‐C in AML cells and thereby decreased translocation of β‐catenin to the nucleus results in a significant down‐regulation of survivin expression at both the mRNA and protein levels. Furthermore, our findings demonstrate that MUC1‐C drives marked increases in survivin levels. The role of survivin as a critical mediator of MUC1 signalling was further demonstrated in an NSG mouse model. Mice inoculated with MUC1 silenced AML cells in which survivin was overexpressed using lentiviral transduction showed greater AML engraftment than mice inoculated with MUC1 silenced AML cells.

Down‐regulation of survivin expression has been shown to overcome drug resistance in acute leukaemia models.^37^, ^49^ Targeting survivin with shRNA in combination with chemotherapy also resulted in the absence of detectable minimal residual disease in a xenograft model of primary leukaemia.^49^ Given these findings, we sought to examine whether MUC1‐C inhibition and the subsequent decrease in survivin levels render AML cells more susceptible to standard chemotherapy. Concurrent treatment of AML cells with the MUC1‐C inhibitor GO‐203 in combination with Ara‐C was found to have a synergistic effect as validated by CompuSyn analysis. These data show that targeting MUC1‐C with GO‐203 can potentially lead to a decrease in anti‐apoptotic properties in AML blasts via survivin down‐regulation, thereby rendering the tumour cells more susceptible to genotoxic damage. A similar effect was demonstrated following MUC1 knockout using CRISPR/Cas9 technology. AML cells lacking MUC1 expression were shown to be significantly more susceptible to Ara‐C‐induced cytotoxic injury. Interestingly, survivin overexpression in MUC1 silenced AML cells led to increased resistance to Ara‐C treatment in AML cells, confirming that MUC1 renders AML more resistant to cytotoxic injury in part via surviving‐mediated mechanism.

Consistent with our results, gene expression analysis of patient with AML demonstrated that lower MUC1 and survivin levels led to significantly prolonged overall survival in patients with complex cytogenetic and molecular abnormalities.

In conclusion, our data show that MUC1‐C signalling is crucial for AML establishment in vivo. MUC1 levels affect the β‐catenin signalling and its downstream target survivin. Furthermore, MUC1‐C inhibition in AML cells with GO‐203 treatment can potentially sensitize drug‐resistant cells to chemotherapeutic regimens via survivin down‐regulation. This combinatorial approach has the potential to eradicate blasts in patients with AML.

## CONFLICTS OF INTEREST

Donald Kufe: Genus Oncology: Consultancy and equity holder.

## AUTHOR CONTRIBUTION

Dina Stroopinsky, Myrna Nahas, Hasan Rajabi, Athalia Pyzer and Maryam Rahimian designed research, performed research and interpreted data. Dina Stroopinsky and Myrna Nahas assisted with manuscript preparation. Turner Kufe, Akriti Kharbanda, Ashujit Tagde, Michal Bar Natan, Maxwell D Coll, Kristen Palmer, Abigail Washington, Leandra Cole, Adam Ephraim and Eleni Anastasiadou performed the research. Salvia Jain and Rebecca Karp Leaf assisted with patient sample acquisition. Donald Kufe designed research, analysed and interpreted data and assisted with manuscript preparation. Jacalyn Rosenblatt and David Avigan assisted with research design, patient sample acquisition, analysed and interpreted data and assisted with manuscript preparation.
