# Supplementary figures and images for: MUC1‐C drives myeloid leukaemogenesis and resistance to treatment by a survivin‐mediated mechanism
Source: J Cell Mol Med. 2018 May 15;22(8):3887–98. doi: 10.1111/jcmm.13662 (PMC6050463; doi:10.1111/jcmm.13662)

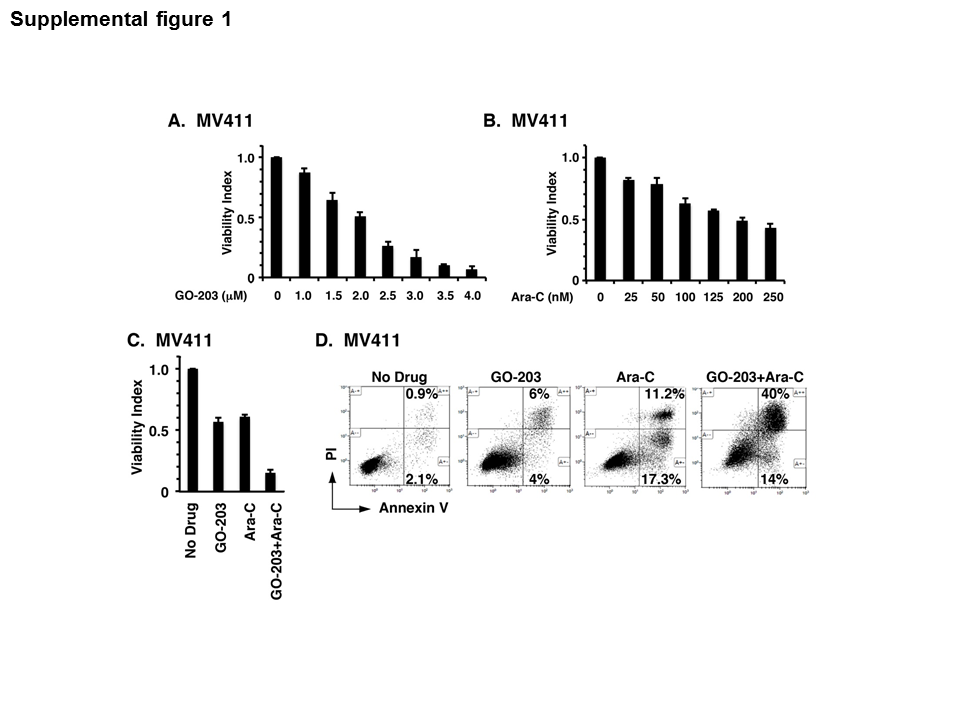

Supplement: Supplementary file 1 [file JCMM-22-3887-s001.tif]

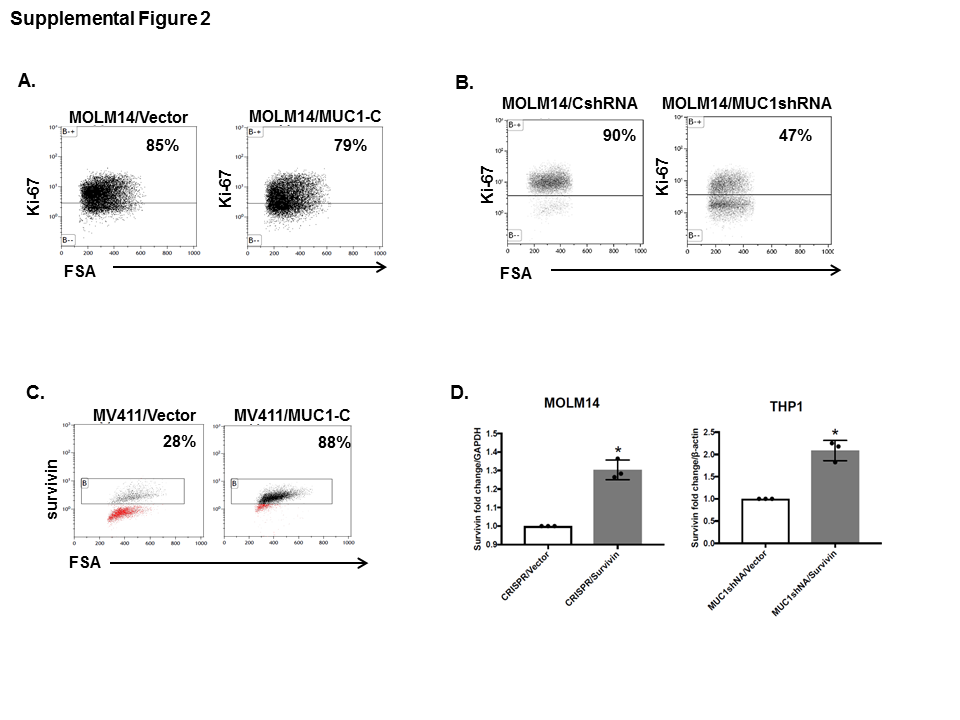

Supplement: Supplementary file 2 [file JCMM-22-3887-s002.tif]
